# Real-world efficacy and safety of letermovir versus ganciclovir prophylaxis in adolescent patients undergoing allogenic hematopoietic stem cell transplantation: a single center observational study

**DOI:** 10.3389/fcimb.2025.1558637

**Published:** 2025-06-03

**Authors:** Ziwei Xu, Xuan Lu, Huafang Wang

**Affiliations:** Institute of Hematology, Union Hospital, Tongji Medical College, Huazhong University of Science and Technology, Wuhan, China

**Keywords:** adolescents, cytomegalovirus, letermovir, ganciclovir, HSCT, hematopoietic stem cell transplant

## Abstract

**Objectives:**

To compare the efficacy and safety of letermovir and ganciclovir for cytomegalovirus (CMV) prophylaxis in adolescent patients (aged 14-17 years) undergoing allogenic hematopoietic stem cell transplantation (allo-HSCT).

**Methods:**

This observational and single-center study collected data from February 2023 and April 2024.

**Results:**

The cumulative incidence of CMV DNAemia following HSCT was 44.4% in the letermovir group (n=20) and 66.3% in the control group (n=32) receiving ganciclovir. Notably, the cumulative incidence of clinically significant CMV infection (csCMVi) was significantly reduced in the letermovir group compared with control patients (11.0% vs 41.3%, p=0.021). Among patients diagnosed with grades II-IV acute graft-versus-host disease (aGVHD), a significantly lower proportion of individuals in the letermovir group presented CMV DNAemia than in the control group (20.0% vs 73.3%, p=0.013). The common adverse events observed in the letermovir group were aGVHD (60.0%), diarrhea (25.0%), and nausea (15.0%). Leukopenia was reported in only one patient, and did not necessitate an adjustment of letermovir dosage.

**Conclusions:**

In this single-center real-world study, letermovir exhibited a favourable efficacy and safety profile for CMV prophylaxis in adolescent patients undergoing HSCT. However, further prospective multi-center studies are warranted to validate our conclusion in adolescent patients.

## Graphical Abstract

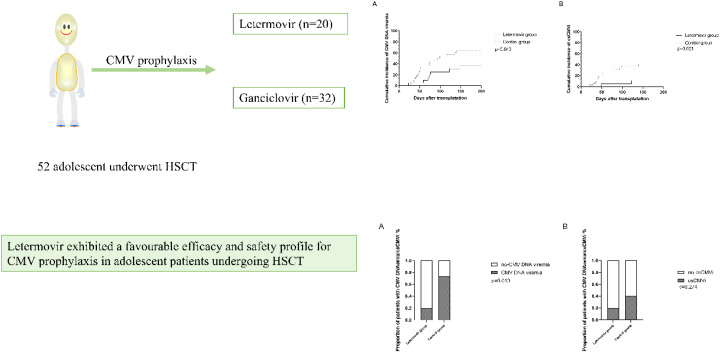

## Introduction

Allogeneic hematopoietic stem cell transplantation (allo-HSCT) represents the cornerstone in the treatment of hematologic disorders. However, the procedure is fraught with significant complications, such as graft-versus-host disease (GVHD) and severe infections like cytomegalovirus (CMV), posing a grave threat to the patient’s prognosis (Teira et al., 2016; Malard et al., 2023).

The CMV serostatus of the donor or recipient emerges as a significant risk factor for CMV reactivation (García-Masedo Fernández et al., 2024; Mehta et al., 2025). The reactivation rate is highest in CMV R+ recipients (50–70%, regardless of donor status), compared to D+/R- recipients (20–25%) (Teira et al., 2016). Furthermore, factors such as the application of antithymocyte globulin (ATG) or alemtuzumab, reduced-intensity conditioning (RIC), umbilical cord blood donor source, and GVHD have also been associated with CMV reactivation (George et al., 2010; Jaing et al., 2019; Heston et al., 2021).

While ganciclovir or valganciclovir has shown efficacy in preventing CMV reactivation, their use is associated with myelosuppression, particularly leukopenia (Ganciclovir prophylaxis of cytomegalovirus infection and disease in allogeneic bone marrow transplant recipients: results of a placebo-controlled, double-blind trial, 1993; Boeckh et al., 2015). This may lead to suspension of CMV prophylaxis, requiring adjustment of immunosuppressive doses (Boeckh et al., 2015). Monitoring of CMV viral load via polymerase chain reaction (PCR) on a weekly basis post-HSCT allows for the detection of CMV DNAemia (Ljungman et al., 2017). Upon reaching a predetermined threshold, preemptive therapy is often initiated, reducing the incidence of CMV disease. However, despite pre-emptive treatment (PET), there is a risk of progression to end-organ disease due to drug failure or resistance, which carries a high morbidity and mortality (Green et al., 2016; Teira et al., 2016).

Letermovir has been approved for CMV prophylaxis in adult patients with CMV seropositive undergoing HSCT (Chen et al., 2018). Letermovir is a novel CMV terminase complex inhibitor that selectively targets the viral pUL56 subunit, thereby blocking the cleavage and packaging of viral DNA into capsids (Boivin et al., 2012). Unlike traditional anti-CMV agents (e.g., ganciclovir), letermovir does not inhibit viral DNA polymerase, which minimizes myelotoxicity—a critical advantage in allo-HSCT recipients (Boivin et al., 2012; Wong et al., 2022; Kleiboeker, 2023). Letermovir has been demonstrated remarkable efficacy in the prevention of CMV infections in adult patients (Körholz et al., 2023; Fukuda et al., 2024; Hopff et al., 2024; Russo et al., 2024). However, letermovir is currently used as an off-label indication in adolescent patients during the study period.

While reports on the efficacy and safety of letermovir in preventing CMV infection in pediatric or adolescent patients undergoing HSCT are scarce, existing evidence suggests that its efficacy and safety profile are comparable to those observed in adults (Körholz et al., 2023; Kuhn et al., 2023; Galaverna et al., 2024). Herein, we present our experience with letermovir as CMV prophylaxis in adolescent patients (aged 14-18 years) undergoing HSCT at our institution.

## Patients and methods

A retrospective cohort study was undertaken to analyze the clinical data of 52 adolescent patients (aged 14-17 years) who underwent allo-HSCT at the Union Hospital of Huazhong University of Science and Technology. The case group (n=20) consisted of adolescent patients who received letermovir for a minimum of 100 days as primary CMV prophylaxis post-HSCT between February 2023 and April 2024, and were followed for more than 100 days except in cases of death. 32 matched control patients received ganciclovir as CMV prophylaxis post-HSCT between September 2022 and August 2023. Informed consent for data collection and analysis was obtained from guardians prior to transplantation, and the study was approved by the institutional review board at the Union Hospital of Huazhong University of Science and Technology.

All patients underwent myeloablative conditioning according to their specific disease type, and the sole source of stem cells was peripheral blood stem cells (PBSCs), which were infused without manipulation following standard mobilization protocols (Xu et al., 2024). The median number of CD34^+^ cells infused was 6.91 × 10^6^/kg, ranging from 3.01× 10^6^/kg to 15.27 × 10^6^/kg, and the median number of nucleated cells infused was 11.44× 10^8^/kg, ranging from 6.21 ×10^8^/kg to 21.70 × 10^8^/kg. ATG was administered from day -4 to day -2, combined with tacrolimus, short-term methotrexate (MTX), mycophenolate mofetil, and anti-CD25 monoclonal antibody (Basiliximab) for mismatched transplants. For HLA-matched donors, patients received cyclosporine, MTX, and Basiliximab for GVHD prophylaxis. Supportive care was provided as previously reported to prevent complications during transplantation (Xu et al., 2024). All patients began receiving CMV prophylaxis after neutrophil engraftment was succeeded until d+100 post-transplant. If aGVHD was not resolved by d+100 in both groups, and patients were receiving corticosteroids or other second-line therapy for aGVHD, CMV prophylaxis was extended. The CMV PCR status of all patients was negative at the time of prophylaxis initiation. All adolescent patients in the study weighed 30kg or more and received a daily dose of 480 mg letermovir. For patients receiving cyclosporine in GVHD prophylaxis, the letermovir dosage was halved. Ganciclovir prophylaxis was administered orally at a dose of 1000 mg three times daily. Neutrophil engraftment was defined as an absolute neutrophil count (ANC) ≥ 0.5 × 10^^9^/L for three consecutive days, and platelet engraftment was defined as a platelet count ≥ 20 × 10^^9^/L for 7 consecutive days, without transfusion. Neutropenia was defined as an absolute neutrophil count ≥1.5 × 10^^9^/L. Thrombocytopenia was defined as a platelet count ≤ 50× 10^^9^/L. Acute GVHD (aGVHD) and chronic GVHD (cGVHD) were graded according to consensus criteria (Sullivan et al., 1991; Przepiorka et al., 1995). PCR for CMV and Epstein-Barr Virus (EBV) DNA in blood was performed twice weekly starting from neutrophil recovery and continuing until day +100. CMV DNAemia was defined as the detection of CMV DNA in the plasma, and the lower limit of detection is 4IU/mL (Ljungman et al., 2017). Patients underwent preemptive therapy when the CMV DNA levels exceeded 400 IU/mL. Clinically significant CMV infection (csCMVi) was defined as the initiation of preemptive therapy or CMV end-organ disease (Marty et al., 2017).

The primary objective of the study was to evaluate the real-world application of letermovir for CMV prevention compared to a historical cohort receiving ganciclovir during the follow-up period. Secondary objectives included assessing the incidence of CMV infection and PET in the two groups, adverse events associated with letermovir, the influence of GVHD treatment on CMV infection, and the risk factors of PET under letermovir prophylaxis.

Baseline characteristics of patients were summarized by frequencies with percentages for categorical variables and median with range for continuous outcomes. The Mann–Whitney U-test was used for continuous variables, and the X^2 test or Fisher exact test were used for categorical data. Overall survival (OS) was defined as the time interval from transplantation until death from any cause and estimated by the Kaplan-Meier method. The cumulative incidence (CI) was calculated using a competing risk model for cGVHD, CMV infections, csCMVi,relapse and transplant-related mortality (TRM). All analyses were performed using SPSS v26.0 and R v 3.5.2.

## Results

### Patient characteristics

The demographic and disease-specific characteristics were succinctly presented in **Table 1**. Each patient underwent only one transplantation during the observation period.

**Table 1:** Baseline characteristics.

Characteristic	Letermovir group (n = 20)	Control group (n = 32)	p value
Age in years; Median (Range)	15 (14–17)	16 (14-17)	0.293
Female gender, n (%)	8 (40.0%)	10 (31.3%)	0.561
Primary underlying disease, n (%)			0.488
Acute myeloid leukaemia	5 (25.0%)	9 (28.1%)	
Acute lymphoblastic leukaemia	11 (55.0%)	12 (37.5%)	
Myelodysplastic syndrome	0	3 (9.4%)	
Aplastic anemia	4 (20.0%)	8 (25.0%)	
Disease Status, n (%)			1.000
Complete remission	18 (90.0%)	28 (87.5%)	
Others	2 (10.0%)	4 (12.5%)	
HLA-matching, n (%)			1.000
Matched unrelated donor	1 (5.0%)	3 (9.4%)	
Matched related donor	6 (30.0%)	9 (28.1%)	
Haploidentical transplantation	13 (65.0%)	20 (62.5%)	
CMV serostatus, n (%)			0.035
D+/R+	12 (60.0%)	22 (68.8%)	
D-/R+	8 (40.0%)	7 (21.9%)	
D+/R-	0	1 (3.1%)	
D-/R-	0	2 (6.3%)	
TBI-based conditioning, n (%)	10 (50.0%)	5 (15.6%)	0.020
ABO mismatch, n (%)			0.857
Match	12 (60.0%)	20 (62.5%)	
Mismatch	8 (40.0%)	12 (37.5%)	
Median CD34^+^ ×10^6^/kg (range)	6.57 (3.23-11.20)	7.13 (3.01-15.27)	0.910
Median TNC ×10^8^/kg (range)	12.56 (6.89-21.70)	10.75 (6.21-20.19)	0.116
GVHD prophylaxis, n (%)			
Tacrolimus+MTX+MMF+Basiliximab+ATG	13 (65.0%)	20 (62.5%)	0.855
CsA+MTX+Basiliximab	7 (35.0%)	12 (37.5%)	

HLA, human leukocyte antigen; CMV, cytomegalovirus; D/R donor/recipient; TBI, total body irradiation; TNC, total nuclear cells; GVHD, graft-versus-host disease; MTX, methotrexate; MMF, mycophenolate mofetil; ATG, anti-thymocyte globulin;CsA, cyclosporine; Chi-square test: Sex, TBI-based conditioning, ABO mismatch, GVHD prophylaxis; Fisher’s exact test: Primary underlying disease, HLA-matching, CMV serostatus, Disease status; Mann-Whitney U-test: Age, Median CD34+ cell count, Median TNC count.

A total of 52 adolescent patients were included in the study, with a median age of 16 years (range, 14-17), comprising 34 males and 18 females. Forty patients underwent HSCT for a malignant disease, with 88.5% of them achieving complete remission (CR) at the time of transplant. All adolescent patients received myeloablative conditioning, with a higher proportion of patients in the letermovir group undergoing total body irradiation-based conditioning compared to the control group (50.0% vs 15.6%, p=0.012). Nineteen patients (36.5%) underwent allo-HSCT with an HLA-matched donor, of whom 4 had matched unrelated donors (MUD) and 15 had matched related donors (MRD). A total of 49 patients (94.2%) were positive for CMV serostatus (D+/R+: 65.4%; D-/R+: 28.8%).

### Engraftment and GVHD

All patients successfully achieved neutrophil and platelet engraftment in both groups. The median time to neutrophil engraftment was 11 days in both groups. The median time to platelet engraftment was 13 days in the letermovir group and 12 days in the control group (p=0.775). As shown in **Table 2**, 12 patients in the letermovir group and 18 patients in the control group developed aGVHD within 100 days post-HSCT. Fifty percent of patients in the letermovir group and 46.9% of patients in the control group were treated with glucocorticoids for the development of grades II-IV aGVHD. The one-year cumulative incidence of cGVHD was comparable between the letermovir group and the control group, with respective percentages 38.8% vs 29.5% (p=0.269).

**Table 2:** Clinical outcomes in the two groups.

Characteristic	Letermovir group (n = 20)	Control group (n = 32)	p
ANC, median (range), days	11 (8-16)	11 (8-14)	0.519
Platelet, median (range), days	13 (9-18)	12 (9-18)	0.775
Outcomes			0.928
Alive, n (%)	17 (85.0%)	27 (84.4%)	
Dead, n (%)	3 (15.0%)	5 (15.6%)	
Cause of death			
Relapse, n (%)	0	2 (6.3%)	
TRM, n (%)	3 (15.0%)	3 (9.4%)	
Acute GVHD, n (%)	12 (60.0%)	18 (56.3%)	1
grades II-IV, n (%)	10 (50.0%)	15 (46.9%)	0.826
Chronic GVHD, n (%)	6 (30.0%)	6 (18.8%)	0.269
Relapse, n (%)	1 (5.0%)	5 (15.6%)	0.387
AML, n (%)	0	1 (3.1%)	
ALL, n (%)	1 (5.0%)	3 (9.4%)	
Others, n (%)	0	1 (3.1%)	
Infection, n (%)			
Bacteria	6 (30.0%)	5 (15.6%)	0.299
Fungi	2 (10.0%)	6 (18.8%)	0.463
EBV	5 (25.0%)	2 (6.3%)	0.092
CMV DNAemia	7 (36.7%)	20 (64.7%)	0.040
csCMVi	2 (11.0%)	13 (41.3%)	0.021

ANC, absolute neutrophil count; TRM, transplantation-related mortality; GVHD, graft-versus-host disease; AML, acute myelogenous leukemia; ALL, acute lymphoblastic leukemia; EBV, Epstein-Barr Virus; CMV, cytomegalovirus; csCMVi, clinically significant CMV infection; Kaplan-Meier method: The median time to neutrophil and platelet engraftment. All other statistical methods for data analysis were Chi-square/Fisher’s exact tests.

### Effectiveness of letermovir as CMV prophylaxis

**Figure 1:**
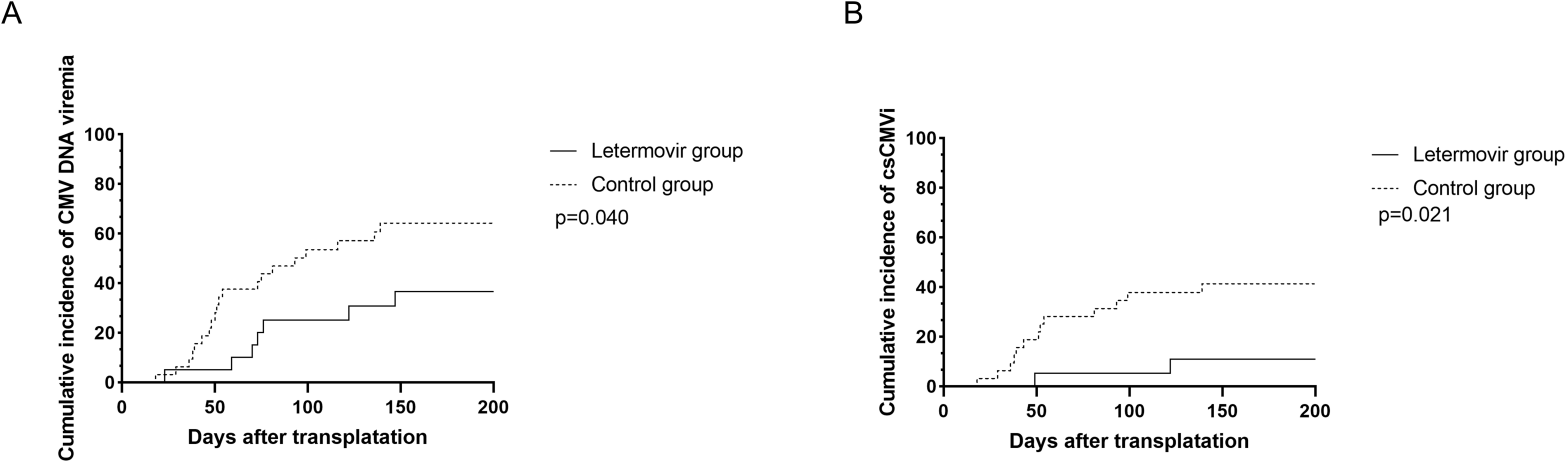
The cumulative incidence of CMV DNAemia **(A)** and csCMVi **(B)** in adolescent patients post-HSCT between letermovir group and control group. Analyses were performed using a competing risk model. Median follow-up was 148 days (range: 23–200) for the letermovir group and 109 days (range: 18–200) for the control group. csCMVi, clinically significant CMV infection; HSCT, allogeneic hematopoietic stem cell transplantation.

**Figure 2:**
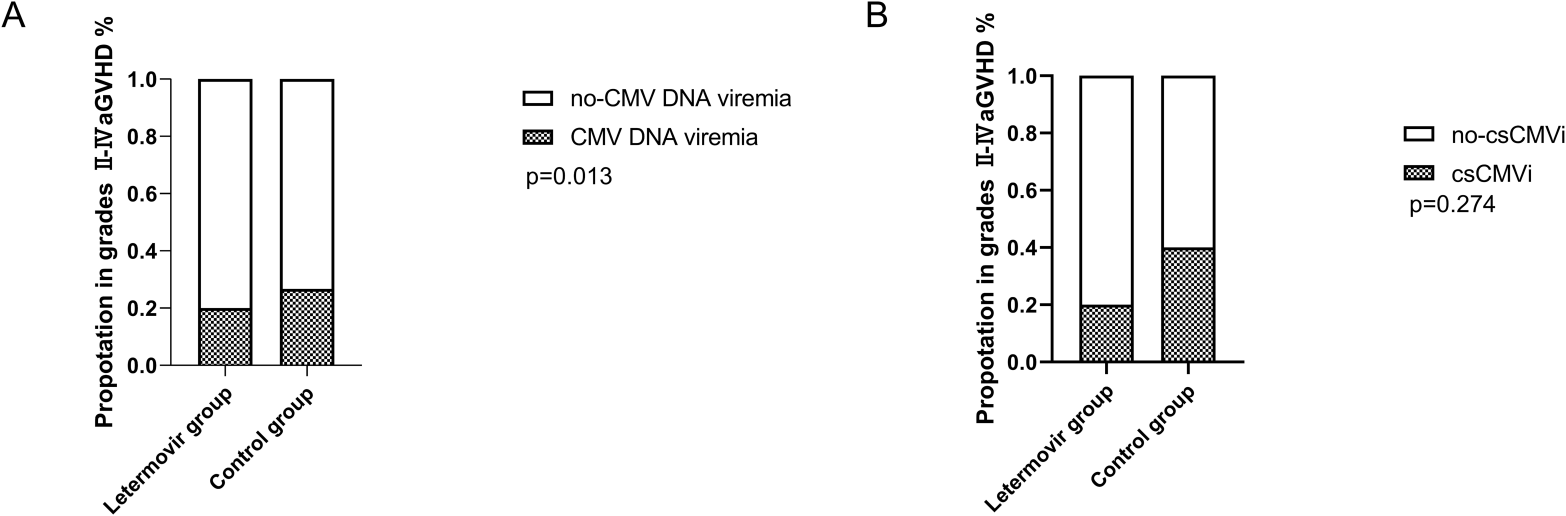
The proportion of CMV DNAemia **(A)** and csCMVi **(B)** in adolescent patients post-HSCT who developed grades II-IV acute GVHD between letermovir group and control group. Analyses were performed with Fisher’s exact test. csCMVi, clinically significant CMV infection; HSCT, allogeneic hematopoietic stem cell transplantation; GVHD, graft-versus-host disease.

All patients who survived beyond 100 days post-transplantation maintained CMV prophylaxis for a minimum duration of 100 days. Eight patients received letermovir prophylaxis extending beyond 100 days (median duration: 150 days; range: 109-189 days), while twelve patients were administered ganciclovir-based prophylaxis with a significantly longer median treatment duration of 173 days (range: 137-201 days). During the observation period, the cumulative incidence of CMV DNAemia at d+200 after HSCT was similar in the group of patients receiving letermovir prophylaxis (36.7%, *95% CI: 33.2-38.2%*, n = 7) compared to control patients (64.7%, *95% CI: 61.7-64.8%*, n = 20), as shown in **Figure 1A** (p=0.040). However, the **Figure 1B** showed that the cumulative incidence of csCMVi was significantly reduced in the letermovir group compared to the control group (11.0%, *95% CI: 9.5-11.6%* vs 41.3%, *95% CI:39.2-42.3%*, p=0.021). Four patients in the letermovir group and 13 patients in the control group received preemptive therapy for CMV DNA levels exceeding 400 IU/mL (**Table 2**). Preemptive therapy typically involved intravenous ganciclovir or foscarnet at the discretion of the clinician. Among patients who developed grades II-IV aGVHD, as showed in **Figure 2A**, a significantly lower proportion of patients in the letermovir group presented CMV DNAemia than in the control group (20.0% vs 73.3%, p=0.013).

As showed in **Figure 2B**, 20.0% and 40.0% of patients in the letermovir group and the control group received preemptive therapy. Preemptive therapy effectively cleared CMV in all patients, and no patients developed CMV end-organ disease.

### Safety

The most common adverse events observed in the letermovir group were aGVHD (60.0%), diarrhea (25.0%), and nausea (15.0%), as shown in **Table 3**. Neutropenia was reported in only one patient and did not necessitate an adjustment of letermovir dosage. However, neutropenia occurred in 6 patients (18.8%) during CMV prophylaxis in the control group, and 4 patients discontinued ganciclovir due to persistent leukopenia. Three patients had a neutrophil count greater than 1×10^^9^/L within one week of stopping the drug, and continued ganciclovir prophylaxis after the neutrophil count exceeded 1.5×10^^9^/L. One patient had a persistently low neutrophil count and therefore received Granulocyte Colony-Stimulating Factor (G-CSF), and foscarnet was used for CMV prophylaxis. Drug-associated thrombocytopenia (platelet count <50×10^9^/L) occurred in 2 patients (10.0%) receiving letermovir versus 9 patients (28.1%) in the ganciclovir group (p=0.112). By day +100 post-transplant, median platelet counts were significantly higher in the letermovir group compared to controls (90×10^9^/L [range: 37-171] vs. 69×10^9^/L [17-114]; p=0.009), suggesting less cumulative marrow toxicity with letermovir prophylaxis (data not shown).

**Table 3:** Adverse events in the safety population.

Adverse event	Letermovir group (n = 20)	Control group (n = 32)	p
Diarrhea	5 (25.0%)	9 (28.1%)	0.534
Nausea	3 (15.0%)	5 (15.6%)	0.637
Neutropenia	1 (5.0%)	6 (18.8%)	0.161
Thrombocytopenia	2 (10.0%)	9 (28.1%)	0.112
Increased creatinine	0	1 (3.1%)	0.615

GVHD, graft-versus-host disease.

### Outcomes and relapse

In total, 8 patients (15.4%) died during the follow-up period. The 1-year cumulative incidence of TRM was 15.0% (95% CI: 13.4–16.3%) in the letermovir group versus 9.4% (95% CI: 8.8–9.9%) in controls (p=0.592). Three patients in the letermovir group died due to transplant-related reasons, two died due to aGVHD, and one died due to life-threatening infections. In the control group, two patients died due to relapse and infection, respectively, and one died due to aGVHD. No patients died due to CMV in either group. The 1-year cumulative incidence of relapse was 5.0% (95% CI: 4.5–5.4%) in the letermovir group and 9.7% (95% CI: 9.1–10.3%) in controls (p=0.548). One patient in the letermovir group and five patients in the control group experienced disease relapse. Critically, no patients in the letermovir group who experienced relapse subsequently died of disease progression; all relapse-associated deaths (n=2) occurred in the control group.

### Discussion

In this single-center real-world study, we retrospectively compared the efficacy and safety profile of letermovir and ganciclovir as CMV prophylaxis in adolescent patients undergoing HSCT. Patients in the letermovir group exhibited a lower cumulative incidence of csCMVi than patients in the control group. However, this finding warrants careful interpretation given the inherent differences in baseline CMV risk profiles between the two cohorts. Notably, the control group included both CMV-seronegative and seropositive recipients, whereas the letermovir group exclusively comprised high-risk CMV-seropositive patients (R+). Previous studies have established that CMV-seronegative recipients have lower risk of CMV reactivation (Heston et al., 2021). Similarly, letermovir demonstrated a safety advantage, particularly with a relatively low incidence of leukopenia. Previous reports have indicated that letermovir has a significant effect in preventing CMV in adults undergoing HSCT (Marty et al., 2017; Fukuda et al., 2024; Russo et al., 2024). In our study, 20 adolescent patients received letermovir, and 32 patients received ganciclovir orally as CMV prophylaxis. The cumulative incidence of CMV DNAemia post-HSCT was similar between the letermovir group and the control group (36.7% vs 64.7%, p=0.040). However, patients in the letermovir group showed a significant reduction in the cumulative incidence of csCMVi compared with patients in the control group (11.0% vs 41.3%, p=0.021). For patients who developed grades II-IV aGVHD, glucocorticoids or other immunomodulatory drugs were necessary to improve the clinical symptoms. Marty et al. indicated that GVHD and the treatment with glucocorticoids resulted in an increased incidence of csCMVi (Marty et al., 2017). In our study, among 25 patients who developed grades II-IV aGVHD, a significantly lower proportion of patients in the letermovir group than in the control group presented CMV DNAemia. No significant difference in the incidence of csCMVi was found between the two groups in our study, which may be related to the limited number of patients studied. Current evidence indicates that gastrointestinal aGVHD, combined drugs such as cyclosporine, carpofonzin, meprednone and other factors affect letermovir valley concentration in patients, which may also affect the clinical efficacy of letermovir in preventing CMV (Qiu et al., 2025). Future tests need to be performed in patients using letermovir, especially in patients with CMV reactivation. Letermovir prophylaxis was associated with delayed CMV immune reconstitution (Moore et al., 2023). This phenomenon may stem from suppressed viral replication during prophylaxis, which limits endogenous antigen exposure necessary for immune priming. Consequently, abrupt cessation of prophylaxis heightens rebound risks, as evidenced by increased csCMVi incidence post-discontinuation (Schleiss, 2021; Zamora et al., 2021).

Daukshus et al. conducted an investigation on letermovir prophylaxis in adolescent allo-HSCT recipients (median age 15.2 years, n=9), demonstrating complete prevention of csCMVi during the prophylactic period (PD et al., 2022). Richert et al. performed a matched retrospective analysis at a single pediatric center, reporting a striking divergence in CMV reactivation outcomes: zero cases occurred in the letermovir cohort versus a cumulative incidence of 61.5% in controls (Richert-Przygonska et al., 2022). The superior prophylactic efficacy against CMV reactivation observed in prior studies compared to our findings may be attributable to differences in letermovir exposure duration (PD et al., 2022; Richert-Przygonska et al., 2022; Chen et al., 2023). Several studies found that the use of letermovir as CMV prophylaxis contributed to improved overall survival and reduced non-relapse mortality in patients receiving HSCT compared with those not receiving letermovir (Mori et al., 2021; Malagola et al., 2024).

A multicenter, randomized, phase 3 trial reported that the incidence of csCMVi was significantly reduced in patients who received extended letermovir prophylaxis (200 days) compared with those who received 100 days (Russo et al., 2024). In our study, eight patients received letermovir for more than 100 days (median, 150 days), and none of these patients developed CMV DNAemia during the follow-up.

Consistent with studies in adults, the most common adverse events with letermovir in children were nausea, vomiting, and mild renal impairment (Richert-Przygonska et al., 2022; Körholz et al., 2023; Kuhn et al., 2023). In general, in our study, letermovir was well tolerated in adolescent patients, and no patient discontinued or reduced the dose due to adverse events. The use of letermovir reduced the proportion of patients with leukopenia compared with the control group. Correspondingly, fewer patients required adjustment of immunosuppressive doses or the use of G-CSF.

Our study has several limitations, such as being a retrospective, single-center study with a limited number of patients. Therefore, future studies are needed to confirm our findings, including prospective, multicenter studies. In addition, this study’s scope did not include CMV genotypic resistance profiling due to institutional constraints in routine resistance testing. Future prospective studies should incorporate UL56/UL89 mutation screening to evaluate potential resistance mechanisms, particularly in breakthrough infections. Our CMV DNAemia data may include non-replicative viral DNA fragments, though clinical correlation with antigenemia and therapeutic interventions suggests biological significance. Future studies incorporating replication-specific markers (e.g., mRNA, DNase-resistant DNA) are warranted. Our study population was aged 14-17, and in the future, we hope to observe the efficacy and safety of letermovir as CMV prophylaxis in pediatric patients under the age of 14.

In conclusion, the single-center real-world study demonstrated that letermovir exhibited favourable efficacy and safety as CMV prophylaxis in adolescent patients undergoing HSCT, compared to ganciclovir. Patients receiving extended duration of letermovir prophylaxis showed a lower incidence of CMV DNAemia. Therefore, further studies are needed in children who receive HSCT to investigate extending the duration of letermovir as a CMV prophylaxis.

## Data Availability

The raw data supporting the conclusions of this article will be made available by the authors, without undue reservation.
